# The Use of Hyaluronic Acid after Tendon Surgery and in Tendinopathies

**DOI:** 10.1155/2014/783632

**Published:** 2014-05-08

**Authors:** Michele Abate, Cosima Schiavone, Vincenzo Salini

**Affiliations:** Department of Medicine and Science of Aging, University G. d'Annunzio, Chieti-Pescara, Via dei Vestini 31, 66013 Chieti, Italy

## Abstract

Viscosupplementation with hyaluronic acid is safe and effective in the management of osteoarthritis, but its use in the treatment of tendon disorders has received less attention. The aim of this review is to summarize the current knowledge on this topic, evaluating experimental and clinical trials. A search of English-language articles was performed using the key search terms “hyaluronic acid” or “viscosupplementation” combined with “tendon,” “tendinopathy,“ “adhesions,“ or “gliding,“ independently. In quite all the experimental studies, performed after surgical procedures for tendon injuries or in the treatment of chronic tendinopathies, using different hyaluronic acid compounds, positive results (reduced formation of scars and granulation tissue after tendon repair, less adhesions and gliding resistance, and improved tissue healing) were observed. In a limited number of cases, hyaluronic acid has been employed in clinical practice. After flexor tendon surgery, a greater total active motion and fingers function, with an earlier return to work and daily activities, were observed. Similarly, in patients suffering from elbow, patellar, and shoulder tendons disorders, pain was reduced, and function improved. The positive effect of hyaluronic acid can be attributed to the anti-inflammatory activity, enhanced cell proliferation, and collagen deposition, besides the lubricating action on the sliding surface of the tendon.

## 1. Introduction

Hyaluronic acid (HA) is an important component of articular cartilage; it is present as a coat around chondrocytes, where it bounds to aggrecan monomers, which imbibe water and are responsible for the resilience of cartilage (i.e., resistance to compression) [1]. Moreover, HA is a major component of the synovial fluid, and, along with lubricin, it is one of the fluid's main lubricating components. The biological activities of HA are very complex: (a) it inhibits matrix metalloproteinases (MMPs) and the phagocytic activity of macrophages and leukocytes; (b) it promotes the release of prostaglandins and the production of tissue inhibitor of MMP-1 and favours the normalization of native hyaluronan synthesis; (c) it acts as free radicals scavenger and stimulates proteoglycans synthesis by chondrocytes; (d) finally, it is provided of protective effects on chondrocytes or cartilage explants from degradation by enzymes [1].

Several clinical trials have shown that viscosupplementation therapy with HA is safe and effective in the management of osteoarthritis (OA) resistant to conventional therapies [2]. This treatment has been approved by Food and Drug Administration for knee osteoarthritis, whereas for the other joints there are very promising results but not conclusive evidence.

Despite the positive results in OA, its use in the treatment of tendon disorders has received less attention. Actually, HA is actively secreted by the tendon sheath and, as for joints, it is an important component of the synovial fluid, which allows a smooth tendon gliding, and provides nutrition to tendon itself [3]. Moreover, it is an important component of tendon structure, being largely present in extracellular space.

The aim of this narrative review is to summarize the current knowledge on this topic, evaluating experimental data and clinical trials. A search of English-language articles was performed in Pubmed, Web of Knowledge (WOK), and EMBASE using the key search terms “hyaluronic acid” or “viscosupplementation” combined with “tendon,” “tendinopathy,” “adhesions,” or “gliding,” independently. Bibliographies were hand-searched to include any applicable studies that were not captured by our search.

Articles were eligible if they provided specific information related to the correlation between hyaluronic acid and tendon disorders.

## 2. Rationale

HA has been mostly used after a surgical procedure for tendon injuries or in the treatment of chronic tendinopathies.

Flexor tendon injuries of the hand are common, especially in the young and working-age population [4]. After surgical repair, the finger motion can be dramatically impaired by oedema, and later by excessive scar formation, and/or by adhesions between the tendon and sheat or other tissues [5]. To limit these negative outcomes, a proper balance between protection and mobilization is required. Indeed, especially in Zone II, a region where the flexor digitorum profundus and superficialis tendons reside within a fibroosseous pulley system, immobilization is useful to allow tendon union but often results in adhesions that impair the physiologic gliding [4]. Postoperative protected mobilization, either active or passive, is usually performed, but early motion may have detrimental effects, contributing to gap formation or tendon rupture, while late mobilization may favour adhesions [6].

In complicated injuries, primary repair of a flexor tendon is often impossible. In these situations, tendon grafts, using extrasynovial tendons (e.g., palmaris longus or plantaris), are often used to reconstruct severely scarred or missing tendons [7]. The extrasynovial grafts have the advantage of being easily harvested, but the higher gliding resistance compared with intrasynovial tendons, due to adhesion formation, represents a main disadvantage on their use [8]. For this reason, physical barriers and biological techniques have been employed to reduce tendon adhesions without adversely affecting the healing process [9, 10]. In this context, HA has been considered as a useful therapeutic tool.

However, in a limited number of cases, HA has been also employed in the treatment of tendinopathies [11–13]. HA is an essential component of the tendon itself. It is well known that, after experimental damage, tendon healing process proceeds along a complex pathway beginning with inflammation and cellular proliferation and followed by tissue formation and maturation, with each phase lasting for days, weeks, and months, respectively [14].

Briefly, in the damaged area, the injury molecular products stimulate an acute inflammatory reaction with the secretion of cytokines, reactive oxygen species, cationic peptides, or proteases. This phase is followed by cell proliferation, collagen and matrix deposition, and tissue remodeling and the final outcome is scar tissue formation, which can partially restore tendon function. Indeed, the mechanical properties are compromised for years, due to composition of the extracellular matrix and its organization.

Basic research has shown that the activity of hyaluronidase 2 is increased in granulation tissue during the healing of equine superficial digital flexor tendon injuries, suggesting that HA plays a relevant role in controlling the healing process in equine tendonitis [15].

## 3. Experimental Studies

Several studies have been performed to evaluate the efficacy of HA on adhesions, gliding resistance, and tendon healing [7–10, 16–21]. Different experimental models have been used: animal species (dog, chicken, rabbit, rat, and horse), tendons (intra- or extrasynovial; auto- or allograft), and procedures of induced tendon damage (surgical, collagenase, or steroid lesion). Moreover, a lot of HA compounds (low and high molecular weight HA, cross-linked HA) have been studied in the form of gel, membrane, or scaffold, administered intra- and peritendinously, both* in vitro* and* in vivo*.

Quite all the authors have shown that HA reduces the formation of scars and granulation tissue and prevents adhesions after tendon repair. The efficacy seems in some way dependent on the HA used. Indeed, the native HA has a limited action because of a very short half-life and a too rapid elimination to serve as a physical barrier between the tendon and the sheath [3]. A slight superiority was found when using the high molecular weight HA (Hylan G-F 20) which has a longer half-life compared to low molecular weight HA [7]. Indeed, in the experiments carried out by Kolodzinskyi et al. [7] on the isolated canine peroneus longus (PL) the gliding resistance of the tendon, treated with Hylan G-F 20, decreased significantly compared to untreated controls.

Other authors have supposed that adhesions could be reduced with more stable HA derivatives and HA could be fixed to the tendon surface, and its removal could be minimized [8–10, 19, 20]. Several HA derivatives have been evaluated.

The carbodiimide-derivatized HA (cd-HA) is less soluble in water than normal HA and therefore has an increased tissue residence time [19, 20]. Preliminary experiments, performed on intact tendon grafts* in vitro*, showed that the preparation improved gliding and that the effect was increased when gelatin was added [8]. Experiments were therefore performed on isolated flexor digitorum profundus tendons in dogs, submitted to dissection, followed by surgical repair. These studies showed that, from the 50th mechanical stress cycle onwards, the gliding resistance was significantly lower in the cd-HA gel group than in the control group, without any difference in the breaking strength nor in tendon stiffness. These results were reported to an increased residence time of HA on the tendon surface, favouring the cross-linking of the HA mixture to tendon proteins [20].

Further studies were carried out by Zhao et al. [18], who studied a compound, characterized by a cd-HA and gelatin, added to lubricin, a mucinous glycoprotein present in the peritendinous fluid. Flexor digitorum profundus tendons from the 2nd and 5th digits of one forepaw of six dogs were transected and repaired. One tendon in each paw was then treated with HA; the other repaired tendon was not treated. Following tendon repair, a forearm cast was applied to fully immobilize the operated forelimb. After 3 and 7 weeks, the dogs were euthanized and the tendons were evaluated. The adhesion formation was assessed in two regions, between the tendon and the sheath and between the tendon and the bone, using a semiquantitative scoring system (none, mild, moderate, and severe). The normalized work of flexion, the gliding resistance, and the maximum breaking force of treated tendons were also recorded. In the HA-lubricin group, the normalized work of flexion was significantly lower, as well as the prevalence of severe adhesions. However, also the maximum breaking force was reduced, suggesting that the positive effects of the compound on adhesions were counteracted by some impairment of tendon healing. The hypothesis was that lubricin could enter the repair site, blocking the adhesion of the healing tendon ends to each other. The authors suggest that a gelatin patch, sealing the tendon and carrying growth factors, could provide a better protection of the lacerated tendon ends from lubricin leakage and enhance the flexor tendon intrinsic healing [21].

Another HA-derived biomaterial is Seprafilm, a bioresorbable membrane composed of chemically modified HA and carboxymethylcellulose [9]. This device, however, suffers from several drawbacks, such as the short* in vivo* residence time and the difficulty to handle when wrapping the tendon, because the material loses its integrity. In contrast, the Carbylan TM-SX (a new carboxylated and thiol-modified HA-derivative) films are elastic, robust, and easy to handle and can maintain their integrity during manipulation. This preparation has been shown to be more effective in reducing the peritendinous adhesions, following partial thickness injury in rabbits, compared to Seprafilm and Carbylan TM-SX sprayable gel [10]. The favourable activity was attributed to the inhibitory activity of thiol-modified HA on cell attachment, spreading, and proliferation, with the compound being negatively charged, hydrophilic, and lacking peptidic epitopes that activate integrins.

The few studies, at present performed in humans in human isolated tendons, confirming experimental research, suggest that tendon surface treatment using HA (cd-HA or added with lubricin) can reduce the excursion resistance in the tendon-pulley unit in the intra- and extrasynovial tendons, facilitating postoperative rehabilitation and improving the clinical outcome [22, 23].

These studies, taken together, suggest that HA diminishes the excursion resistance after tendon repair and can be useful to prevent adhesion until the synovial surface is fully developed.

Less experimental work has been performed to assess whether HA can be effective in tendon healing [15, 17]. In Achilles tendonitis, induced by repeated injections of corticosteroids in rats, the effects of Hylan G-F 20 were investigated by means of histopathology [17]. For the tendon, thickness, staining affinity, nuclear appearance, and fibrillar appearance were taken into account, whereas, for paratenon fibrosis and oedema, capillary changes and inflammation were assessed, according to a semiquantitative scoring system. Compared to controls, injected with saline, Hylan-injected tendons and paratenons showed significantly lower scores, especially after 75 days. These positive effects were attributed mainly to the anti-inflammatory activity of the agent, which inhibits, both* in vitro* and* in vivo*, the leukocyte function (phagocytosis, adherence, and mitogen-induced stimulation). Moreover, it was supposed that HA, according to studies in chicken embryos and in endothelial cell cultures, could inhibit vasculogenesis, explaining the reduced vascular score at the calcaneal insertion [17].

The positive role of HA in tendon healing has been confirmed by the effects of the Seprafilm antiadhesion membrane, composed of sodium-hyaluronate plus glucosamine HCl-chondroitin sulfate [30]. In rabbits, submitted to tendon rupture and ensuing surgical repair, after 84 days, the treatment significantly enhanced the maturation rate of the tenoblasts, fibrillogenesis, the diameters of the collagen fibrils, and fibrillar density. These findings suggest that Seprafilm antiadhesion membrane could be effective in restoring the morphological properties of injured superficial digital flexor tendon of rabbits and might be helpful for future clinical trial studies in tendon ruptures.

## 4. Clinical Trials

Experimental evidence suggests that, in the clinical setting, it may be possible to inject HA within the flexor sheath during the postoperative period in patients at risk for adhesions or with noteworthy oedema.

In a multicenter randomized controlled trial, Hyaloglide, a thick and sticky gel which adheres to the surface of the tendon, was applied after flexor tenolysis in Zone II following failed flexor tendon repair [4]. 30, 60, 90, and 180 days after surgery, patients in the study group, compared with not treated controls, showed a greater total active motion and finger function, with an earlier return to work and daily activities. Noteworthy, Hyaloglide did not affect tendon and wound healing and did not increase the complications rate.

Similarly, Özgenel and Etöz [5] investigated the efficacy of high molecular HA injections versus placebo (saline) on functional outcomes after Zone II flexor tendon repair. Three doses were given (one at the time of tenorrhaphy and two at one-week intervals) around the tenorrhaphy site, and a rehabilitation program was started on the 3rd postoperative day. Range of motion (active and passive) and functional outcome (Strickland classification) were assessed. In the short term (3 weeks), no difference between the two groups was observed; however, at 3 months and in the long term, a significant increase in the total values of the passive and active range of motion was present in fingers treated with HA. Noteworthy, no adverse events (signs of inflammation or tendon rupture) were seen in any cases.

Besides the prevention of adhesions after flexor tendons repair, the therapeutic efficacy of HA has been evaluated in other tendon diseases.

In an open-label randomized study, patients with trigger finger were randomly divided into two groups: (1) HA plus corticosteroid and (2) open surgical release [11].

At 6 and 12 months, 93% of patients of the first group had complete symptom resolution, compared to 73% submitted to surgery.

In 50 athletic patients with patellar tendinopathy (stage 2 or 3 according to Blazina's classification), a mixture of 25 mg HA and 1 mL of 1% lidocaine was injected blindly at the proximal interface between the posterior surface of the patellar tendon and the infrapatellar fat pad or into the region of maximum tenderness [12]. A week after the first injection, other injections were done on the patient's request; conservative treatments (exercises and instrumental therapies) were also prescribed. An average of 2 injections/case (range 1–11; total 135) was performed with 12-month (range 3–49 months) interval between injections. After treatment, 54% of patients were rated in excellent conditions (return to previous athletic activities), while 40% in good conditions complained of some degree of limitation. The open design and the subjective evaluation methods used (no imaging) are important limitations of this study.

Petrella et al. [13] determined the efficacy of periarticular HA injections in patients suffering from tennis elbow. In this prospective randomized clinical trial, 165 subjects with chronic lateral epicondylosis received 2 blind injections of 1.2 cc of HA (once a week) into the subcutaneous tissue and muscle, from the lateral epicondyle toward the primary point of pain. Saline solution was injected in the control group (166 patients). Pain, both at rest and after grip testing, was significantly reduced in the study group compared to controls after 30, 90, and 365 days. The treatment was highly satisfactory and resulted in earlier return to pain-free sport.

The experience of HA use in rotator cuff tendinopathies is more consistent [24–29]. Patients with different rotator cuff diseases (full or partial thickness tear, tendinosis) were treated with HA injections and compared to placebo or active treatments (steroids or physical therapies). The main results of these trials are summarized in Table 1. A superior therapeutic effect was observed in comparison to placebo, but no significant difference was shown when steroids or physical therapy was used as controls.

## 5. Conclusions

Experimental research and pivotal clinical trials suggest that HA is effective in preventing adhesions after flexor tendons surgery. Different preparations and procedures are at present under study to define the best option.

Promising results have been also observed in the treatment of tendinopathies. In general terms, the positive effect relies on the anti-inflammatory activity of HA, enhanced cell proliferation, and collagen deposition, besides the lubricating action on the sliding surface of the tendon. However, it must be underlined that in the majority of studies the drug was not injected inside the degenerated tendon, but nearby and/or into the articular space. It can be speculated that the modifications of the synovial fluid can exert a positive effect on the tendon itself, but it cannot be excluded that the clinical improvement may secondary to the positive action on osteoarthritis frequently associated.

## Figures and Tables

**Table 1:** Effects of HA in rotator cuff disorders.

Author (year)	Disease	Experimental groups^∧^	Protocol	Outcomes measures	Follow-up	Results
Shibata et al. (2001) [24]	FTT	LMW HA (27) Steroid (28)	5 intra-articular injections (1-week interval)	ROM, UCLA	6 months	No difference

Meloni et al. (2008) [25]	Tendinosis	LMW HA (28) Saline (28)	5 injections over the superior tendon surface (1-week interval)	ROM, VAS	12 months	Better results in HA group

Costantino et al. (2009) [26]	FTT	LMW HA (22)* No control group	3 intra-articular injections (1-week interval)	ROM, VAS, Costant	6 months	Pain reduction ROM improvement

Chou et al. (2010) [27]	PTT Tendinosis	LMW HA (25) Saline (26)	5 injections into SAD (1-week interval)	VAS, Costant	33 months	Better result in HA group

Ozgen et al. (2012) [28]	Tendinosis	HMW HA (12)* Physical therapy (12)*	3 intra-articular injections (1-week interval)	ROM, VAS	3–42 months	No difference

Merolla et al. (2013) [29]	Tendinosis	LMW HA (25) Rehab (23)	2 injections over the superior tendon surface (2-week interval)	VAS, Costant, Oxford	6 months	No difference at week 2 ; HA better at weeks 4, 12, and 24

FTT: full thickness tear.

PTT: partial thickness tear.

^∧^In brackets the number of patients is reported.

*Home exercise programs were prescribed.

SAD: subacromial-deltoid bursa.

## References

[1] Abate M, Pulcini D, di Iorio A, Schiavone C (2010). Viscosupplementation with intra-articular hyaluronic acid for treatment of osteoarthritis in the elderly. *Current Pharmaceutical Design*.

[2] Rutjes AW, Jüni P, da Costa BR, Trelle S, Nüesch E, Reichenbach S (2012). Viscosupplementation for osteoarthritis of the knee: a systematic review and meta-analysis. *Annals of Internal Medicine*.

[3] Hagberg L, Heinegard D, Ohlsson K (1992). The contents of macromolecule solutes in flexor tendon sheath fluid and their relation to synovial fluid; A quantitative analysis. *Journal of Hand Surgery*.

[4] Riccio M, Battiston B, Pajardi G (2010). Efficiency of hyaloglide in the prevention of the recurrence of adhesions after tenolysis of flexor tendons in zone II: a randomized, controlled, multicentre clinical trial. *Journal of Hand Surgery: European Volume*.

[5] Özgenel GY, Etöz A (2012). Effects of repetitive injections of hyaluronic acid on peritendinous adhesions after flexor tendon repair: a preliminary randomized, placebo-controlled clinical trial. *Ulusal Travma ve Acil Cerrahi Dergisi*.

[6] Corradi M, Bellan M, Frattini M, Concari G, Tocco S, Pogliacomi F (2010). The four-strand staggered suture for flexor tendon repair: in vitro biomechanical study. *Journal of Hand Surgery*.

[7] Kolodzinskyi MN, Zhao C, Sun YL (2013). The effects of hylan g-f 20 surface modification on gliding of extrasynovial canine tendon grafts in vitro. *Journal of Hand Surgery*.

[8] Momose T, Amadio PC, Zobitz ME, Zhao C, An K-N (2002). Effect of paratenon and repetitive motion on the gliding resistance of tendon of extrasynovial origin. *Clinical Anatomy*.

[9] Işik S, Oztürk S, Gürses S (1999). Prevention of restrictive adhesions in primary tendon repair by HA-membrane: experimental research in chickens. *British Journal of Plastic Surgery*.

[10] Liu Y, Skardal A, Shu XZ, Prestwich GD (2008). Prevention of peritendinous adhesions using a hyaluronan-derived hydrogel film following partial-thickness flexor tendon injury. *Journal of Orthopaedic Research*.

[11] Callegari L, Spanó E, Bini A, Valli F, Genovese E, Fugazzola C (2011). Ultrasound-guided injection of a corticosteroid and hyaluronic acid: a potential new approach to the treatment of trigger finger. *Drugs in R and D*.

[12] Muneta T, Koga H, Ju YJ, Mochizuki T, Sekiya I (2012). Hyaluronan injection therapy for athletic patients with patellar tendinopathy. *Journal of Orthopaedic Science*.

[13] Petrella RJ, Cogliano A, Decaria J, Mohamed N, Lee R (2010). Management of Tennis Elbow with sodium hyaluronate periarticular injections. *Sports Medicine, Arthroscopy, Rehabilitation, Therapy and Technology*.

[14] Andia I, Sanchez M, Maffulli N (2010). Tendon healing and platelet-rich plasma therapies. *Expert Opinion on Biological Therapy*.

[15] Foland JW, Trotter GW, Powers BE, Wrighley RH, Smith FW (1992). Effect of sodium hyaluronate in collagenase-induced superficial digital flexor tendinitis in horses. *American Journal of Veterinary Research*.

[16] Tatari H, Skiak E, Destan H, Ulukuş Ç, Özer E, Satoğlu S (2004). Effect of hylan G-F 20 in achilles’ tendonitis: an experimental study in rats. *Archives of Physical Medicine and Rehabilitation*.

[17] Tuncay I, Ozbek H, Atik B, Ozen S, Akpinar F (2002). Effects of hyaluronic acid on postoperative adhesion of tendo calcaneus surgery: an experimental study in rats. *Journal of Foot and Ankle Surgery*.

[18] Zhao C, Sun YL, Kirk RL (2010). Effects of a lubricin-containing compound on the results of flexor tendon repair in a canine model in vivo. *The Journal of Bone & Joint Surgery*.

[19] Yang C, Amadio PC, Sun Y-I, Zhao C, Zobitz ME, An K-N (2004). Tendon surface modification by chemically modified HA coating after flexor digitorum profundus tendon repair. *Journal of Biomedical Materials Research B: Applied Biomaterials*.

[20] Sun Y-L, Yang C, Amadio PC, Zhao C, Zobitz ME, An K-N (2004). Reducing friction by chemically modifying the surface of extrasynovial tendon grafts. *Journal of Orthopaedic Research*.

[21] Jones ARC, Flannery CR (2007). Bioregulation of lubricin expression by growth factors and cytokines. *European Cells and Materials*.

[22] Akasaka T, Nishida J, Imaeda T, Shimamura T, Amadio PC, An K-N (2006). Effect of hyaluronic acid on the excursion resistance of tendon graft: a biomechanical in vitro study in a modified human model. *Clinical Biomechanics*.

[23] Taguchi M, Zhao C, Sun Y-L, Jay GD, An K-N, Amadio PC (2009). The effect of surface treatment using hyaluronic acid and lubricin on the gliding resistance of human extrasynovial tendons in vitro. *Journal of Hand Surgery*.

[24] Shibata Y, Midorikawa K, Emoto G, Naito M (2001). Clinical evaluation of sodium hyaluronate for the treatment of patients with rotator cuff tear. *Journal of Shoulder and Elbow Surgery*.

[25] Meloni F, Milia F, Cavazzuti M (2008). Clinical evaluation of sodium hyaluronate in the treatment of patients with sopraspinatus tendinosis under echographic guide: experimental study of periarticular injections. *European Journal of Radiology*.

[26] Costantino C, Olvirri S (2009). Rehabilitative and infiltrative treatment with hyaluronic acid in elderly patients with rotator cuff tears. *Acta Biomedica de l’Ateneo Parmense*.

[27] Chou W-Y, Ko J-Y, Wang F-S (2010). Effect of sodium hyaluronate treatment on rotator cuff lesions without complete tears: a randomized, double-blind, placebo-controlled study. *Journal of Shoulder and Elbow Surgery*.

[28] Ozgen M, Fırat S, Sarsan A, Topuz O, Ardıç F, Baydemir C (2012). Short- and long-term results of clinical effectiveness of sodium hyaluronate injection in supraspinatus tendinitis. *Rheumatology International*.

[29] Merolla G, Bianchi P, Porcellini G (2013). Ultrasound-guided subacromial injections of sodium hyaluronate for the management of rotator cuff tendinopathy: a prospective comparative study with rehabilitation therapy. *Musculoskeletal Surgery*.

[30] Menderes A, Mola F, Tayfur V, Vayvada H, Barutçu A (2004). Prevention of peritendinous adhesions following flexor tendon injury with seprafilm. *Annals of Plastic Surgery*.

